# Cryptic invasion of a parasitic copepod: Compromised identification when morphologically similar invaders co-occur in invaded ecosystems

**DOI:** 10.1371/journal.pone.0193354

**Published:** 2018-03-14

**Authors:** M. Anouk Goedknegt, David W. Thieltges, Jaap van der Meer, K. Mathias Wegner, Pieternella C. Luttikhuizen

**Affiliations:** 1 NIOZ Royal Netherlands Institute for Sea Research, Department of Coastal Systems, and Utrecht University, AB Den Burg Texel, The Netherlands; 2 Alfred Wegener Institute, Helmholtz Centre for Polar and Marine Research, Germany; Stockholm University, SWEDEN

## Abstract

Despite their frequent occurrence and strong impacts on native biota, biological invasions can long remain undetected. One reason for this is that an invasive species can be morphologically similar to either native species or introduced species previously established in the same region, and thus be subject to mistaken identification. One recent case involves congeneric invasive parasites, copepods that now infect bivalve hosts along European Atlantic coasts, after having been introduced independently first from the Mediterranean Sea (*Mytilicola intestinalis* Steuer, 1902) and later from Japan (*Mytilicola orientalis* Mori, 1935). At least one report on *M*. *intestinalis* may have actually concerned *M*. *orientalis*, and *M*. *orientalis* thus qualifies as a “cryptic invader”. Because these two parasitic copepods are morphologically similar, knowledge about their distribution, impact and interactions depends crucially on reliable species identification. In this study, we evaluated the reliability of morphological identification of these two species in parts of their invasive range in Europe (Dutch Delta and Wadden Sea) in comparison with molecular methods of well-established accuracy based on COI gene sequences and ITS1 restriction fragment length polymorphism. Based on seven easily measured or scored macro-morphological variables that were recorded for 182 individual copepods isolated from blue mussels (*Mytilus edulis* Linnaeus, 1758*)*, principal component analysis showed two relatively distinct but overlapping morphological species groups for females, but no clear separation in males. Discriminant function analysis showed that the females can be discriminated reasonably well based on some of the morphological characteristics (identification error rate of 7%) while males cannot (error rate of 25%). The direction of the dorsolateral thoracic protuberances was identified as the most important trait for species discrimination, but among the morphological features checked, none could flawlessly discriminate between both species. We recommend the use of molecular techniques in future studies of invasive *Mytilicola* to reliably discriminate between the species. The morphological similarity of these two invaders suggests a more general problem of cryptic invasions and compromised identification of parasites in invaded ecosystems. This problem should be borne in mind whenever invasive parasites are investigated.

## Introduction

Biological invasions represent a well-known threat to ecological communities worldwide [1–4], yet it is not uncommon for the presence of an invasive species to remain undetected for longer periods of time [5]. One reason for this is that because of morphological similarity, invasive species might be mistaken for native species or other introduced species that have invaded the same localities [5–9]. While such cryptic invasions have occasionally been revealed for free-living species [5–9], cryptic invasions of internal parasites are more problematic to detect as the organisms are hidden inside their hosts.

The invasion of two congeneric parasites infecting the intestines of marine bivalve species along the northwestern European coast might represent such a case of a cryptic invasion of parasites. The parasitic copepod *Mytilicola intestinalis* Steuer, 1902 was first described in the mussel *Mytilus galloprovincialis* Lamarck, 1819 in the Adriatic Sea [10] and presumably spread with infected mussels as fouling on ships’ hulls to the Atlantic coast of Europe, where it started to infect native bivalve species [11–14]. The congeneric *Mytilicola orientalis* Mori, 1935 was first documented in Pacific oysters (*Magallana* (previously *Crassostrea*) *gigas* (Thunberg, 1793)) from Japan [15] and was introduced to British Columbia in 1938 in shipments of Pacific oysters [16]. Via subsequent oyster transports to France in the 1970s [17], *M*. *orientalis* spread northward with its host to the Dutch Delta [18] and the Dutch, German and Danish Wadden Sea [19, 20]. The two copepod species have accumulated a minimum interspecific genetic difference of 16.08% while showing very low intraspecific diversity for COI (cytochrome-*c*-oxidase I, a mitochondrial gene) [19]. Their divergence time could thus be in the order of seven to eleven million years ago based on a molecular clock commonly used for crustaceans [21, 22], and their status as distinct species by molecular criteria is not in dispute.

The first morphological species description for *M*. *intestinalis* by Steuer in 1902 [10] was later followed by a more detailed characterization by the same author [23]. When *M*. *orientalis* was later discovered in Japan, its investigator Mori was aware of the existence of *M*. *intestinalis* and included a list of differences between the taxa in the original species description [15]. These differences were: 1) more prominent dorsolateral thoracic protuberances in the Japanese species, 2) no dorsolateral protuberances on the first thoracic body segment (males only), and 3) upper lip triangular with a small notch on the tip instead of being rounded with a wavy edge. This last feature has not been mentioned again in later literature, presumably because it required a magnification of 270× and the complicated removal of other mouth parts to be visible [15], and is thus unsuitable in practice for screening large numbers of individuals. In a later study, Ho and Kim in 1992 [24] mention other features that discriminate between the species: 4) longer ovisacs in *M*. *orientalis* (females only), and 5) parallel or nearly parallel caudal rami in *M*. *orientalis* versus diverging caudal rami in *M*. *intestinalis*. Perhaps because two of the five adduced discriminated characters are sex-specific, and one requires intensive microscopy preparation, subsequent researchers have typically focussed on the dorsolateral thoracic protuberances and caudal rami for species identification [18, 19]. Since Ho and Kim [24] no new morphological research has been published on these two taxa to the best of our knowledge.

In practice, these small interspecific differences between the two copepods make a correct morphological species assessment difficult, and this likely led researchers to draw erroneous conclusions about the distribution and host range of both *Mytilicola* species in the past [19]. For instance, a study in the Exe estuary in the UK reported the presence of *M*. *intestinalis* in Pacific oysters [25], even though this parasite-host relationship has never been reported again after the existence of *M*. *orientalis* in these waters became known and it may have been an error [19, 26]. The lack of successful artificial infections of Pacific oysters with *M*. *intestinalis* supports this [19] (personal communications of M. E. Feis). Potential misidentifications may also underlie the often contradictory results of studies on the impacts of the two parasite species on ecologically or commercially important bivalve hosts e.g., [27, 28]. When the presence of only a single species of parasite continues to be assumed by researchers, the introduction of a second copepod species in northwestern Europe qualifies as a cryptic invasion *sensu* [4].

Currently, the two copepod species do not only co-occur along the Atlantic coasts of northwestern Europe, but they also have overlapping host ranges, both reportedly being capable of infecting blue mussels (*M*. *edulis*) [18–20, 26, 29], common cockles (*Cerastoderma edule* (Linnaeus, 1758)) [26, 27, 30] and Pacific oysters (*M*. *gigas*) [25, 31, 32]. However, whether the latter species actually serves as a host for *M*. *intestinalis* is debatable, as was discussed above [19, 26]. The sympatric occurrence of both copepods in the invaded region while sharing at least two host species increases the chance of misidentification even more.

The aim of this study was to assess the reliability of morphology-based identification of *M*. *intestinalis* and *M*. *orientalis* in co-invaded ecosystems. We approached this in three ways. First, we made quantitative morphological measurements of a large pool of *Mytilicola* spp. from blue mussel and Pacific oyster hosts originating from different locations in the Dutch and German Wadden Sea and the Dutch Delta, where the geographical ranges of the two parasite species overlap. In this invaded region, prevalences and mean intensities (± SE) of *M*. *intestinalis* (3–72%, 2.4 ± 0.3) and *M*. *orientalis* (3–63%, 2.1 ± 0.2) in blue mussels and *M*. *orientalis* in oysters (2–43%, 4.1 ± 0.6) can be high [26]. Second, we verified the species identity of every copepod molecularly by applying a diagnostic RFLP assay (Restriction Fragment Length Polymorphism) [26]. And third, we applied multivariate morphometric analyses to investigate the accuracy of morphological identification and to identify reliable features for species discrimination. In addition, as various methods of preservation and storage are known to modify or destroy morphological characteristics [33, 34], we investigated the effect of several preservation and storage methods on the morphology of both species.

## Materials and methods

### Parasite sampling

**Fig 1:**
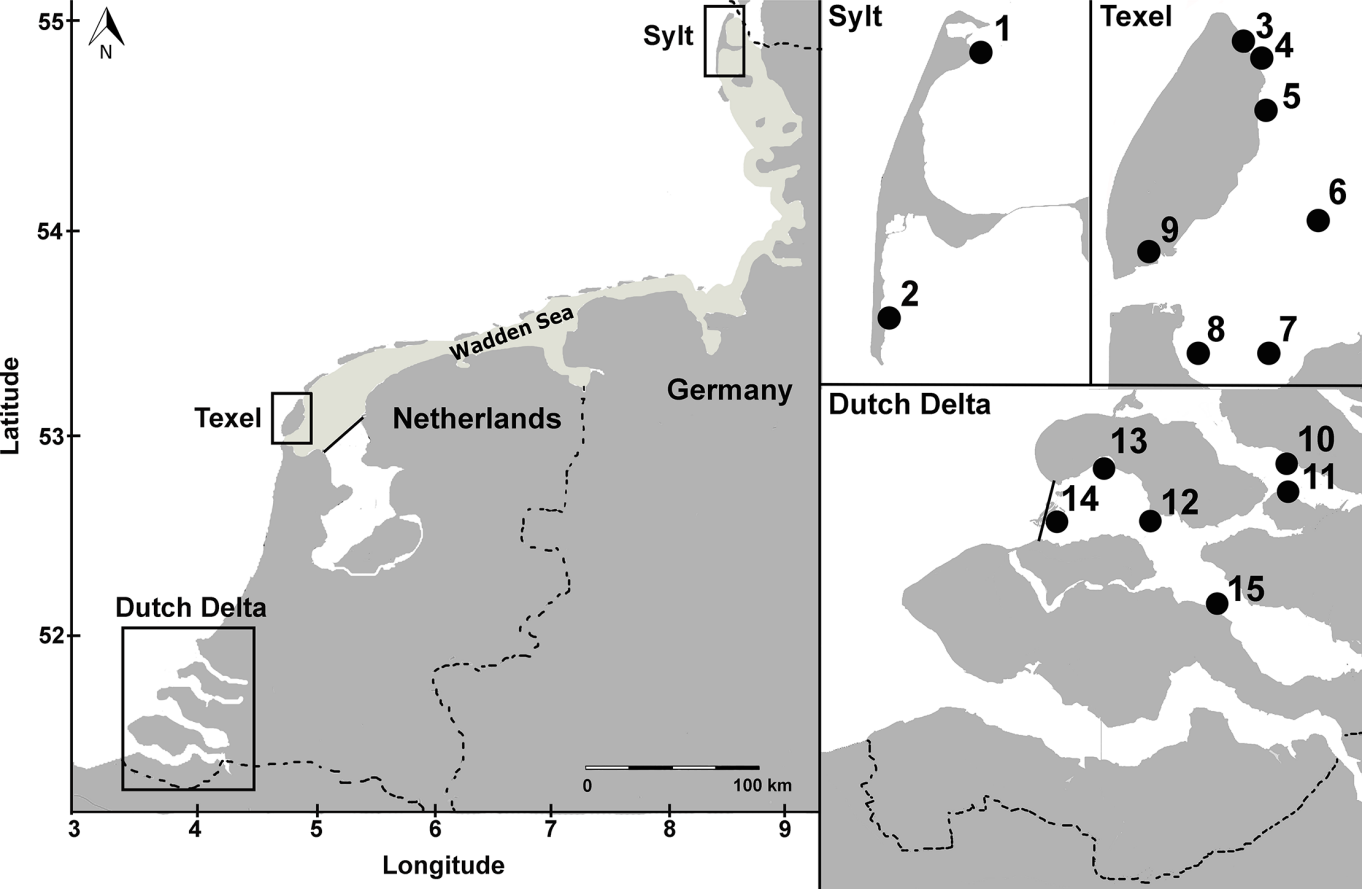
Sampling locations of blue mussel (*Mytilus edulis*) and Pacific oyster (*Magallana* (previously *Crassostrea*) *gigas*) hosts. Left: The sampled regions in the Dutch Delta and the Wadden Sea (shaded area), with the islands Sylt (north) and Texel (south). Above right: Sampling locations around the islands of Sylt and Texel in the Wadden Sea. Below right: Sampling locations in the Dutch Delta. For exact coordinates see S1 Table.

To ascertain whether morphological identification based on macro-characteristics is reliable for all *Mytilicola* individuals found in the introduced region, we used a large pool of specimens originating from different locations and different host species. Copepods were collected in two regions that were important invasion pathways for both parasites: the Dutch Delta (6 locations; May 2012) and the Wadden Sea (9 locations; different months in 2010–2012; Fig 1; S1 Table). A *Natuurbeschermingswet* (Nature Conservation Law) license was obtained for the field work in the Netherlands. For the German Wadden Sea samples, permission came from Nationalparkamt Schleswig-Holsteinisches Wattenmeer, Tönning. The field work did not involve endangered or protected species and complied with local legislation.

Before dissecting specimens of the two main hosts, Pacific oysters (*Magallana gigas*; n = 109, mean shell length ± SE: 120.3 mm ± 2.8 mm) and blue mussels (*Mytilus edulis*; mean shell length ± SE: n = 198, 47.3 mm ± 0.6 mm), we measured the maximum length of each shell with Vernier calipers to the nearest 0.1 mm. Subsequently, the shells were opened, and the tissue was searched for the presence of *Mytilicola* individuals by using a magnification glass (magnification 3–8×). All *Mytilicola* individuals were removed from their hosts and stored in 96% ethanol. Individual copepods were then randomly drawn from each region/host species combination until, when possible, all host-sex categories (i.e., males and females of *M*. *intestinalis* and males and females of *M*. *orientalis*; see ‘Morphological scoring and identification’ section below) were filled with at least 20 individuals for the Dutch Delta and 25 individuals for the Wadden Sea, where we sampled more locations (Fig 1, S1 Table). However, as expected, *M*. *intestinalis* was not found in Pacific oysters and therefore this host/parasite category remained empty.

### Morphological scoring and identification

**Fig 2:**
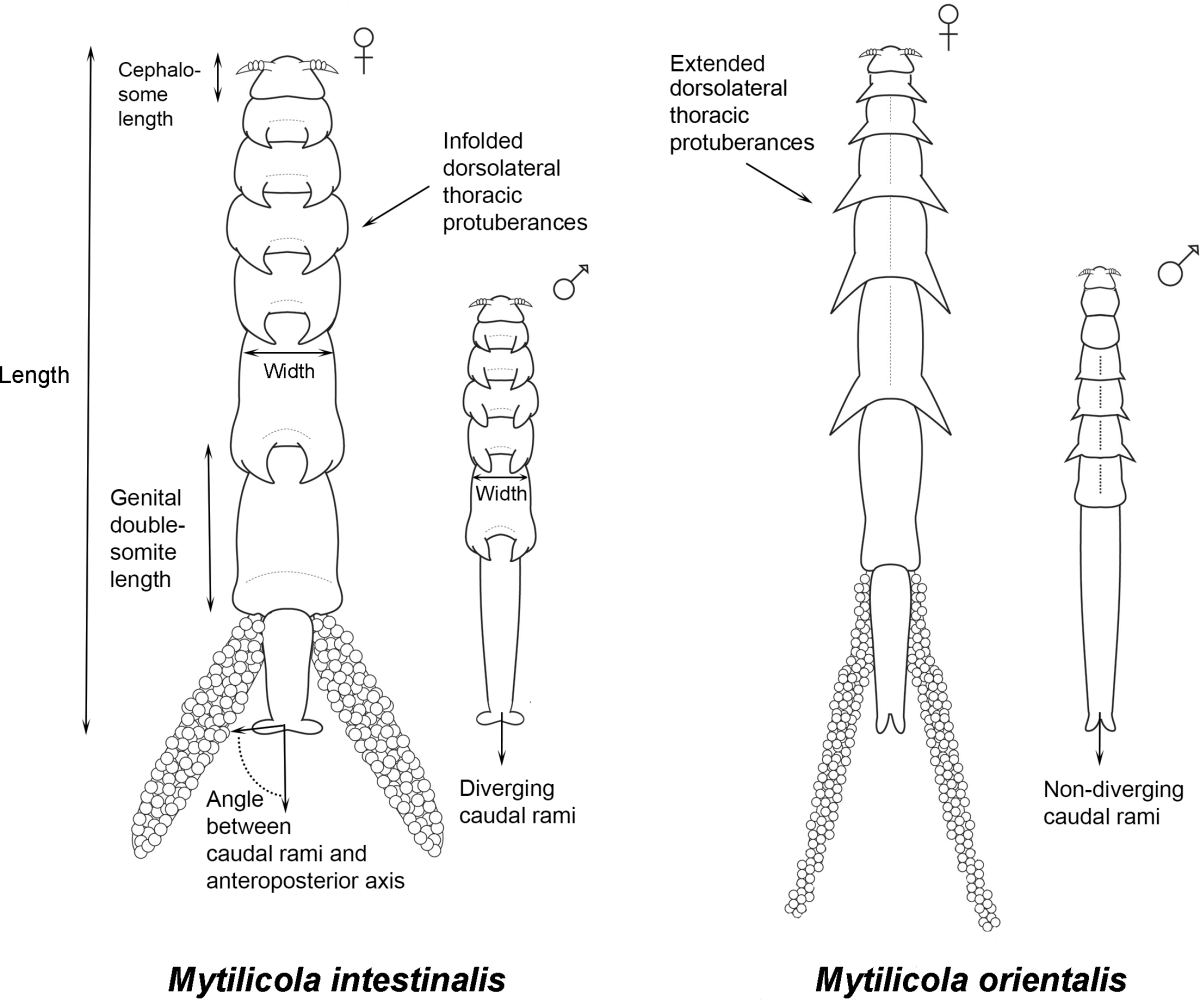
Schematic representation (not to scale) of both sexes of both introduced *Mytilicola* species. On the left both sexes of *M*. *intestinalis* and on the right both sexes of *M*. *orientalis*, all viewed from the ventral side, with indications of the body size measurements that were taken. Note the dorsolateral thoracic protuberances which are folded inwards in *M*. *intestinalis* and extended outwards in *M*. *orientalis*. For detailed drawings see original species descriptions, including views from other sides and close-ups [15, 23]. Drawings with courtesy of Felipe Ribas.

Morphological species identification of individual copepods (performed by MAG) was based on the direction of the dorsolateral thoracic protuberances (folded inwards in *M*. *intestinalis* and extended outwards for *M*. *orientalis* [15, 19] and on the shape of the caudal rami (thick and diverging in *M*. *intestinalis* and narrow and non-diverging in *M*. *orientalis*, Fig 2) [19]. Regarding sex determination, individuals were considered to be females when egg sacs were present and, if not, when the cephalosome was trapezoidally shaped [24] and the maxilliped (referred to as the second maxilliped in early descriptions [15, 23]) was missing (too small to be shown in Fig 2) [10]. Additionally, only in females the genital double-somite was trapezoidal (Fig 2) (MAG, unpublished observations).

After pre-sorting the copepods into the four species-sex categories the same observer (MAG) made quantitative morphological observations of them. See Fig 2 for more details on body measurements of both sexes of both parasite species. With a camera (AxioCam ICc3) attached to a stereo microscope (Zeiss V8 discovery), pictures were taken of each individual copepod, filling the field of vision view as much as possible. The magnification used was 10× for females and 30× for males. Body size measurements were done using the software package AxioVision. Cephalosome length (maximum), body width (males: thinnest part of the animal; females: between the 4^th^ and 5^th^ metasomal body segments) and body length were measured, and the error rate (± SE) based on ten repeated length measurements was 0.40 ± 0.09 μm for males and 0.50 ± 0.13 μm for females. The divergence of the caudal rami [19] was measured as the angle between the caudal rami and the anteroposterior axis (see Fig 2). In addition, the number of body segments carrying dorsolateral thoracic protuberances was counted and the direction of the protuberances was noted (folded inwards or extended outwards). For females, the length of the genital double-somite was measured. With this set of macro-characters, we covered all the previously published traits that are typically used to distinguish between the two species [10, 15, 18, 19] as well as features related to their general shape characters. Shape of the upper lip was not observed in this study, although it supposedly differs between the species [15], because to do so would require detailed microscopical work, which is unpractical (and has, to our knowledge, never been applied) in large field studies. We ignored other microscopic details, such as the number of body segments carrying dorsolateral thoracic protuberances and the shape of the first and second antennae, because no variation or difference was originally [10, 15] nor subsequently noted for these characters.

### Molecular identification

For the molecular identification of each copepod individual we used two assays, one targeting the mitochondrial cytochrome-c-oxidase I gene (COI) and one for the nuclear internal transcribed spacer 1 (ITS1). The combination of both assays potentially allowed us to identify hybrids with the ITS assay, and the direction of hybridisation with the COI assay.

For the COI assay, we newly developed two taxon-specific primers. They were designed on the basis of previously published *Mytilicola intestinalis* and *M*. *orientalis* sequences (Genbank accession numbers HM775191-HM775197) [19]. The new primers (MOICOIf 5'-CTTAATTACAGGGGTMTGATCGG-3' and MOICOIr 5'-TCGATCTGTTAAAAGCATAGTAATYG-3') amplify a 534 bp fragment yielding a PCR product of 583 bp (base pairs) in length.

A diagnostic RFLP (restriction fragment length polymorphism) assay was developed that could distinguish between *M*. *intestinalis* and *M*. *orientalis*. The restriction enzyme NciI recognizes and cuts the sequence CC/SGG (where S = C or G and / indicates the cut site). The PCR product of *M*. *intestinalis* is cut once by NciI, and that of *M*. *orientalis* is cut twice. In *M*. *intestinalis* this results in two restriction fragments that are 366 and 217 bp long. In *M*. *orientalis* the resulting pattern consists of three fragments, respectively 286, 222 and 75 bp long. This difference between the species was visualized on 2% agarose gels, which were run for 120 min. at 60 V, followed by staining with ethidium bromide. The COI assay was performed on all morphologically identified individuals (n = 307).

For the ITS assay we developed a TaqMan assay [29] targeting a species-discriminating single nucleotide polymorphism. We amplified the target region with the primers IntOri_ITS_F 5'-GCGTGTCGGAATGTGAACTG-3' and IntOri_ITS_R 5'-CCTGAGCCAGACATGGACAGA-3' and discriminated the species with the probes Myt_ITS_intP 5'FAM-ACATATGAGCATCGACAATTTGAACGC-3'BHQ1 and Myt_ITS_oriP 5'HEX-AGCATCGACACTTTGAACGCATATTGC-3'BHQ1. Each 20 μl reaction consisted of 10 μl of TaqMan Fast PCR Master Mix (Applied Biosystems, Darmstadt, Germany), 2 μl of each 5μM primer, 1 μl of each 5μM probe, 3 μl of nuclease-free water and 1 μl of sample DNA. An ABI 7500 fast system (Applied Biosystems, Darmstadt, Germany) was used for amplification and end point detection by applying the following cycling program of 60 s at 60°C and 20 s initial denaturation at 95°C followed by 40 cycles of 3 s denaturation at 95°C and 30 s combined annealing and extension at 60°C before a final post-PCR stage of 60 s at 60°C. This assay could successfully discriminate between the pure species and also detect potential hybrids (simulated by mixed DNA) and was performed on a subset of 75 individuals originating from Texel.

### Effects of preservation and storage method on morphology

To investigate the effects of preservation and storage method on copepod macro-morphology, we sampled males and females of both *Mytilicola* species from blue mussel hosts (mean ± SD: 50.0 ± 6.3 mm) which were collected at two locations at the island of Texel: Vlakte van Kerken and Mokbaai (locations 5 and 9, respectively, in Fig 1). These samples were randomly assigned to two groups: 1) samples taken fresh from the host, measured, stored in 96% ethanol and measured again after two weeks (n = 33); and 2) samples taken fresh from the host, measured, frozen (-20°C) for six weeks, defrosted, measured, stored in 96% ethanol and measured again after two weeks (n = 29; for descriptions of morphological measurements see the ‘Morphological scoring and identification’ section above).

### Statistical analysis

All statistical analyses were performed using the statistical software package R [35]. Homogeneity and normality assumptions were checked by using boxplots, histograms and qqplots [36].

#### Effects of origin

In order to rule out any potential biases of the origin of the parasite on body measurements, we used linear models to investigate the relationships between parasite body size and 1) host species (only for *M*. *orientalis* which infects both host species), 2) host size (for each of three parasite-host combinations) and 3) region (for each of the two host species). Sex of the parasite was used as an additional factor in all models. Parasite intensity was included as covariate to the host size models. In the region models, we corrected parasite body size for host size by using the residuals of a linear regression of host size and parasite size as response variable, and the molecular identity of the parasite species (only for the mussel model) and region as explanatory variables. We used parasite body length for these investigations, because this variable was correlated with other size variables (cephalosome length, body width and length of the genital double-somite, the latter only for females; see S1 Fig).

#### Multivariate morphometric analyses

As both parasite species only co-occur in blue mussels in the investigated region, only parasites originating from this shared host were used in the multivariate analyses. The morphology of each individual parasite was characterized as described above via seven morphometric parameters: body length, cephalosome length, body width, angle between caudal rami and anteroposterior axis, number of body segments and direction of dorsolateral thoracic protuberances (see ‘Morphological scoring and identification’ section above), in which the latter was a dummy-coded nominal variable. The variable ‘genital double-somite’ is a feature only of females, resulting in a total of seven different morphological variables for females and six for males. For this reason and the fact that sex was only assessed morphologically, the multivariate morphometric analyses were executed for each sex separately.

These analyses were performed in two steps. First, a principal component analysis (PCA) was conducted to illustrate the relative contribution of each of the morphological variables for each parasite species. Prior to this analysis, all the morphometric variables were checked for outliers, which were removed when the value was larger than the mean plus three times the standard deviation, and subsequently all morphometric variables were log-transformed. Second, a discriminant function analysis (DFA) was performed to investigate the accuracy of the morphological species identifications. With this analysis, the hypothesis was tested that the *a priori* defined groups of parasites (molecular species identity) would differ significantly in their combination of morphometric variables. In the DFA, untransformed variables (excluding outliers) were standardized by using the decostand function in the vegan package in R [37]. The DFA was executed by the lda function of the MASS package [38], in which the Wilks lambda statistic was used to test for an overall group effect. To determine the most important variables in the discrimination between groups, a stepwise discriminant function analysis was employed. Based on the total sum of Mahalanobis distances, this backward selection procedure removed variables to produce a discriminant function with only important variables. In addition to the analysis of the full morphological data set, we did several straightforward calculations on the proportions of correct identifications when using only the two morphological features that are the easiest to assess (angle between caudal rami and anteroposterior axis; and direction of the dorsolateral thoracic protuberances).

#### Effects of preservation and storage method

Differences between body size measurements within group 1 (fresh → ethanol samples) were tested with (paired) Welch’s t-tests when variables were normally distributed and with non-parametric Wilcoxon signed-rank tests when this assumption was violated. Differences between measurements within group 2 (fresh → frozen → ethanol samples) were tested with a repeated-measures ANOVA. To test for differences between groups for both fresh and ethanol treatments, Welch’s t-tests or non-parametric Wilcoxon signed-rank tests were used.

## Results

### Molecular results

Of the 307 morphologically identified *Mytilicola* specimens (Table 1), most individuals displayed one of the two expected banding patterns in the PCR product and could accordingly be assigned to one of the two *Mytilicola* species (for examples of banding patterns see S2 Fig). Nevertheless, in 15 cases, banding patterns deviated from expectations in three different ways. The PCR fragment of one individual remained uncut after the restriction reaction, eight individuals displayed an ambiguous pattern consisting of bands from both species (e.g., lane 15 in S2 Fig) and six individuals showed incompletely digested patterns (e.g., lanes 1–8 and lane 16 in S2 Fig). All these individuals were omitted from subsequent analyses. This resulted in a pool of 292 molecularly identified individual copepods of both sexes that originated from three different regions and two different host species, as follows: *M*. *intestinalis* female (n = 40), *M*. *intestinalis* male (n = 47), *M*. *orientalis* female (n = 109) and *M*. *orientalis* male (n = 96). The TaqMan assay was performed on a subset of 75 individuals all originating from Texel. In all 68 cases for which both the TaqMan assay and the COI assay successfully amplified, the species identification was identical, and no hybrids were detected.

**Table 1:** Sample sizes of introduced *Mytilicola* species in different sub-categories.

Host species	Parasite species	Sex	Dutch Delta	Wadden Sea	Total
***Magallana gigas***	*M*. *orientalis*	Males	22	31	53
		Females	24	32	56
***Mytilus edulis***	*M*. *orientalis*	Males	20	27	47
		Females	21	35	56
	*M*. *intestinalis*	Males	20	30	50
		Females	19	26	45
	Total		126	181	307

### Effects of origin

#### Host species and size

**Fig 3:**
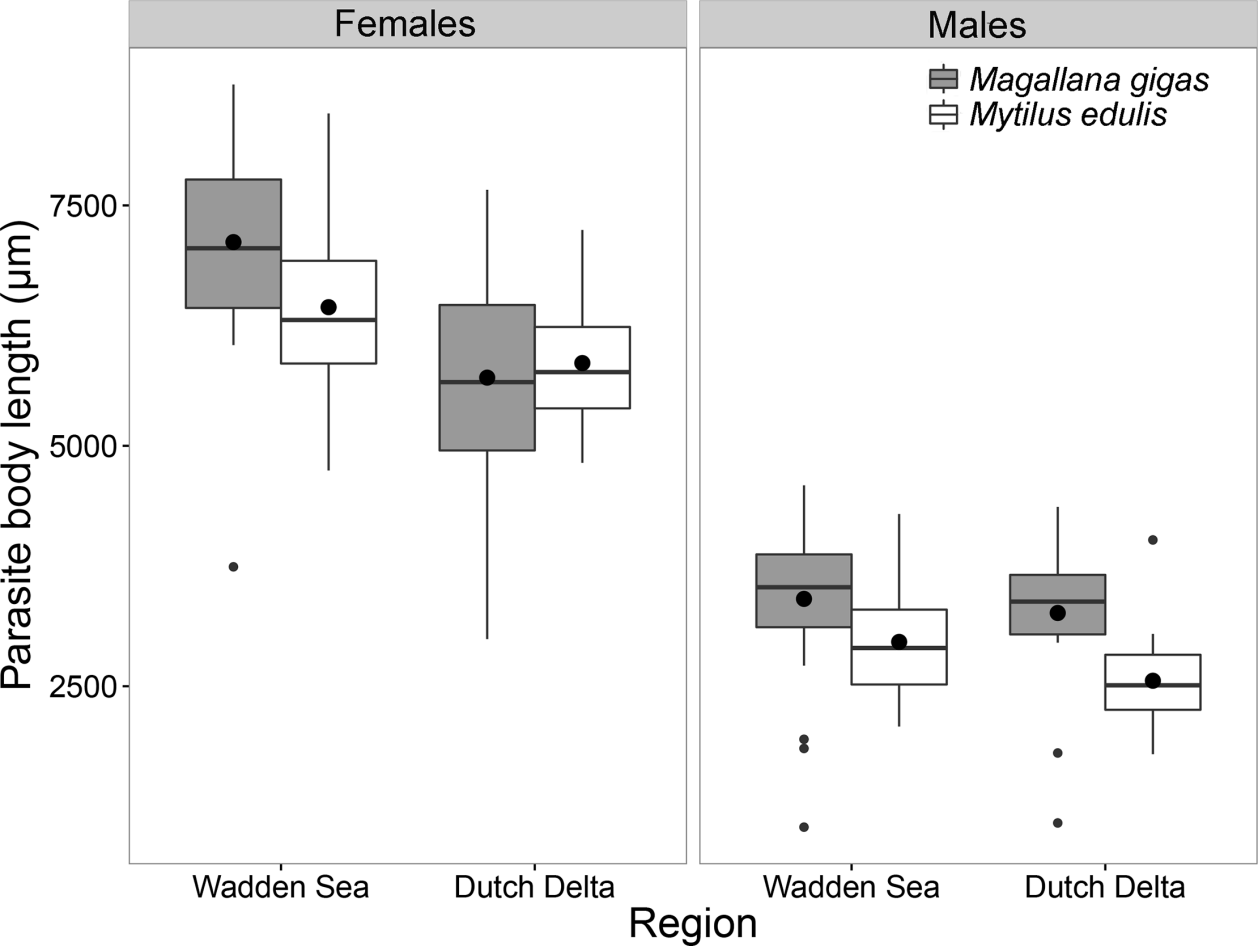
Boxplots of *Mytilicola orientalis* body length (μm) in both host species. Female (left) and male (right) copepods originating from oysters (*Magallana gigas*) in grey and from mussel (*Mytilus edulis*) hosts in white, from the Dutch Delta and Wadden Sea. The boxes represent the interquartile range, the whiskers denote the lowest and highest values within the 1.5 interquartile range, the black line in each box denotes the median, the large black dots represent the mean and the smaller dots outside the boxes are outliers.

For *Mytilicola orientalis*, which uses both blue mussels (*Mytilus edulis*) and Pacific oysters (*Magallana gigas*) as hosts, host species identity was an important determinant for parasite body length as the copepods were significantly larger in oysters (linear model; F_1,193_ = 6.721, p < 0.05; Fig 3). Additionally, in both host species, females of *M*. *orientalis* larger than males (F_1,193_ = 599.470, p < 0.001).

**Fig 4:**
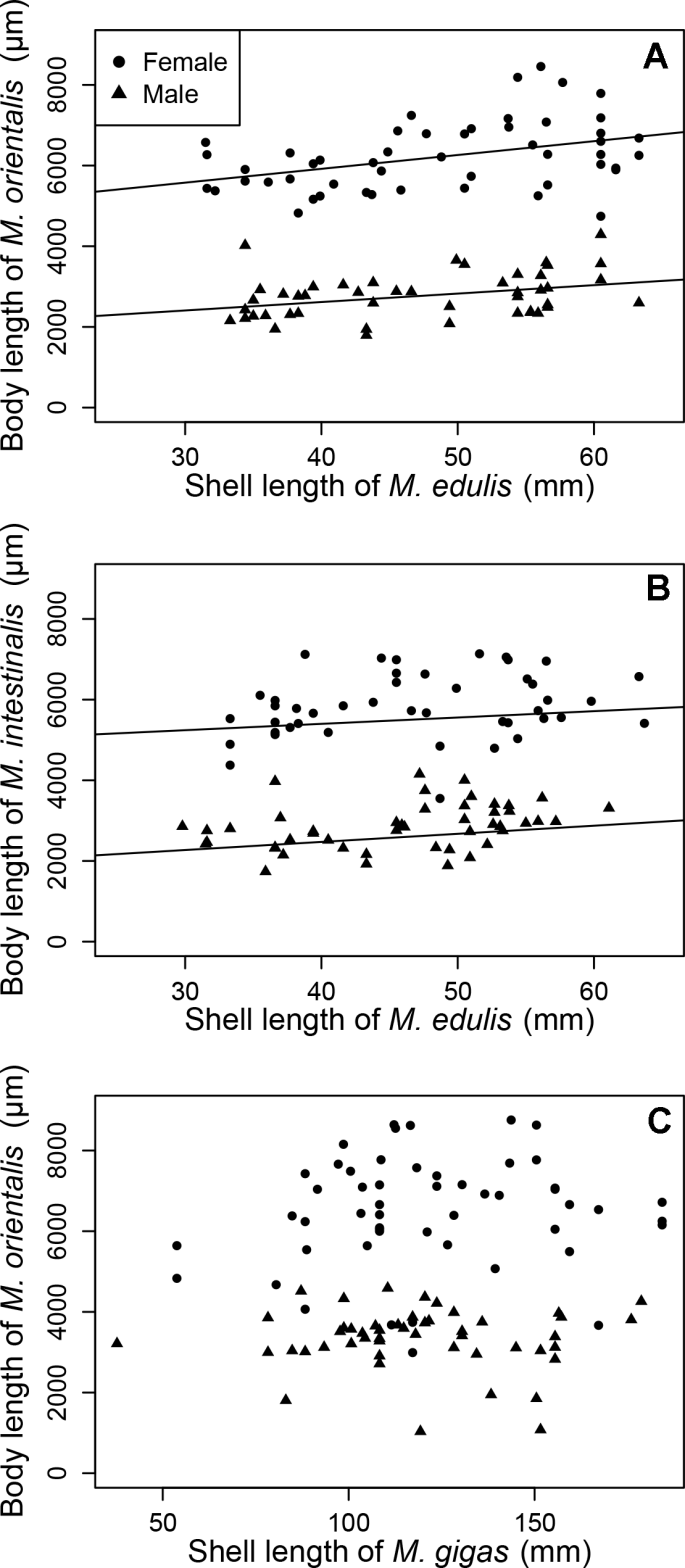
Relationship between host shell length (mm) and parasite body length (μm) per sex for each *Mytilicola*-host species combination. (A) *M*. *orientalis* in blue mussels (*Mytilus edulis*). (B) *M*. *intestinalis* in blue mussels. (C) *M*. *orientalis* in Pacific oysters (*Magallana gigas*). Fitted lines are significant regressions.

Parasite body length was positively related to host size in mussels (linear regression; *M*. *orientalis* R^2^ = 0.87, p < 0.001; *M*. *intestinalis* R^2^ = 0.85, p < 0.01), but not in oysters (p = 0.149; Fig 4). In addition, parasite intensity was positively related to body length of *M*. *intestinalis* in mussels (linear regression, F_1,86_ = 59.754, p < 0.01), but not for *M*. *orientalis* in either mussels (linear regression, p = 0.846) or oysters (p = 0.181). For all combinations of parasite species and hosts in this model, there was a significant difference between the parasite sexes regarding the relationship between host size and parasite body length (*M*. *orientalis* in mussels F_1,91_ = 598.653, p < 0.001; *M*. *intestinalis* in mussels F_1,86_ = 453.474, p < 0.001, *M*. *orientalis* in oysters F_1,96_ = 201.696, p < 0.001). No significant interaction between sex and host size was found for any of the parasite-host combinations (*M*. *orientalis* in mussels p = 0.366; *M*. *intestinalis* in mussels p = 0.787; *M*. *orientalis* in oysters p = 0.376).

#### Geographical region

**Fig 5:**
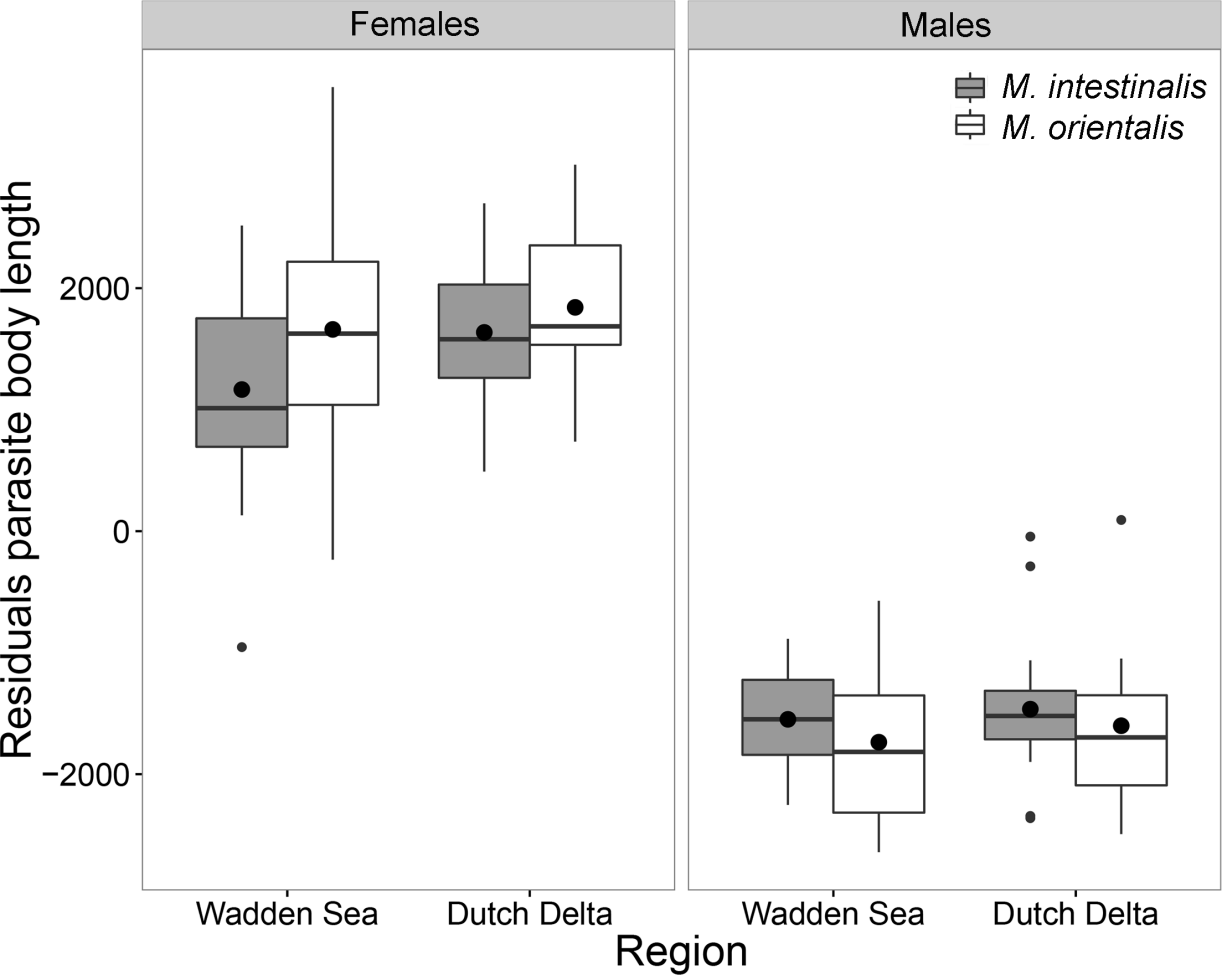
Parasite body length (corrected for host size using linear regression) for both introduced *Mytilicola* species. **Females (left) and males (right) of** *M*. *intestinalis* (grey) and *M*. *orientalis* (white) in each surveyed region. The boxes represent the interquartile range, the whiskers denote the lowest and highest values within the 1.5 interquartile range, the black line in each box denotes the median, the large black dots represent the mean and the smaller dots outside the boxes are outliers.

Body length of both parasite species (corrected for host size) in blue mussels did not differ between regions (p = 0.350; Fig 5). Furthermore, within mussel hosts, *M*. *orientalis* was overall larger than *M*. *intestinalis* (F_1,179_ = 15.568, p < 0.001), but a significant interaction term with sex showed that males of *M*. *orientalis* males were smaller than those of *M*. *intestinalis* males (F_1,179_ = 7.110, p < 0.01). Furthermore, for both parasite species, females were larger than males (F_1,179_ = 1048.029, p < 0.001).

In Pacific oysters, *M*. *orientalis* were larger in the Wadden Sea than in the Dutch Delta (F_1,97_ = 9.656, p < 0.01) and females were larger than males (F_1,97_ = 240.112, p < 0.001). In addition, a significant interaction term between region and sex (F_1,189_ = 9.297, p < 0.01), showed that males in the Dutch Delta are slightly larger than males in the Wadden Sea, while for females this is the opposite.

### Reliability of morphological identification

#### Principal component analysis

**Fig 6:**
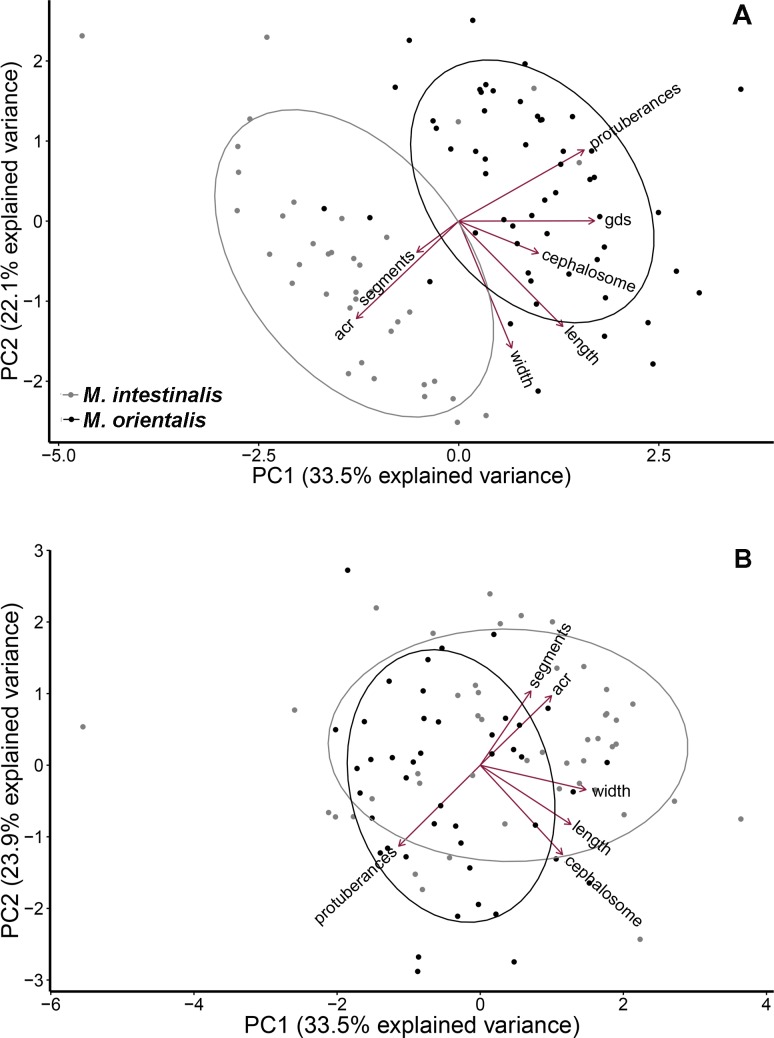
Principal component analysis of *Mytilicola* for each sex separately. (A) Females (n = 92; seven morphological variables). (B) Males (n = 90, six variables). In grey, individual parasites that were molecularly identified as *M*. *intestinalis*; in black, similarly identified *M*. *orientalis* (gds = length of the genital double-somite; acr = angle between caudal rami and anteroposterior axis).

Morphological differences between parasite species, as visualized by Principal Component Analysis (PCA), showed limited overlap between *Mytilicola* species for females (n = 92, Fig 6A), but considerable overlap in males, as many individuals presented intermediate morphologies (n = 90, Fig 6B). This sex-related difference in species partitioning had consequences for the assessment of the reliability of morphological species identification for both sexes using Discriminant Function Analysis (DFA).

#### Discriminant function analysis for females

In total, 92 females of *Mytilicola* were entered into the DFA with the following prior probabilities per group: *M*. *intestinalis* 0.41 (n = 38) and *M*. *orientalis* 0.59 (n = 54). One discriminant function was extracted, and this was highly significant (Wilks’ λ = 0.227, F_1,141_ = 40.925, p = < 0.001), indicating a clear discrimination between groups. The first discriminant function with standardized discrimination coefficients for females is given by (gds = length of the genital double-somite; acr = angle between caudal rami and anteroposterior axis):

$$F_{i}=1.33\times protuberances_{i}+0.62\times gds_{i}+0.21\times segments_{i}+0.03\times cephalosome_{i}-0.55\times acr_{i}-0.07\times length_{i}-0.02\times width_{i}$$

**Fig 7:**
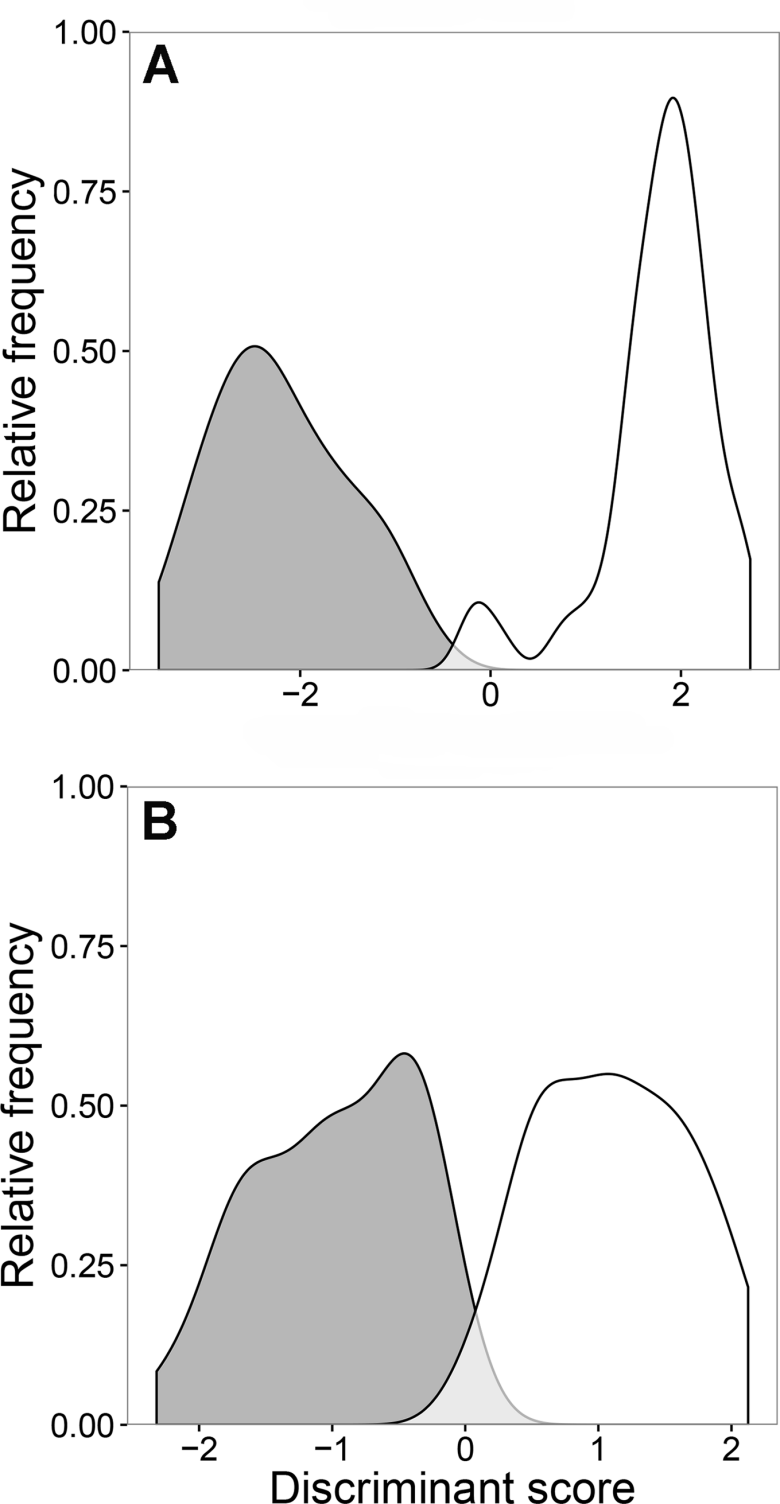
Discriminant distributions of *Mytilicola* for each sex separately. (A) Females (n = 92) and (B) males (n = 90) of the parasitic copepods *M*. *intestinalis* (grey) and *M*. *orientalis* (white). The x-axis is the discriminant score of the first discriminant function and the y-axis is the relative frequency of the observations. The light grey area indicates “mistaken” assignments in comparison with molecular identifications.

The discriminant distributions showed minimal overlap between the two groups, an indication that the discrimination was relatively accurate (Fig 7A). The classification of the groups was highly successful for females; 95% of molecularly identified *M*. *intestinalis* females and 91% of *M*. *orientalis* females were correctly assigned to the respective *Mytilicola* groups.

The morphological features used for identification strongly differ in their importance for a correct assignment. One variable, the direction of the dorsolateral thoracic protuberances, had by far the highest coefficient in the discriminant function. Similarly, the backward selection procedure showed that the direction of the dorsolateral thoracic protuberances, the length of the genital double-somite and the angle between caudal rami and anteroposterior axis were the last variables to be dropped, indicating their importance in discriminating between the two parasite species.

When using only the two morphological features that were the easiest to assess (the direction of the dorsolateral thoracic protuberances and the angle between caudal rami and anteroposterior axis), a large proportion of individuals could still be correctly assigned to the genetically identified species. By using the former character alone, 97% of the *M*. *intestinalis* females and 87% of the *M*. *orientalis* females were correctly identified whereas use of the latter character alone resulted in correct identification of 81% of the *M*. *orientalis* females and 84% of the *M*. *intestinalis* females. An angle of 20° between the caudal rami and the anteroposterior axis was judged to be the cut-off point based on the group averages, < 20° being a “narrow” angle (*M*. *intestinalis*) and ≥ 20° being a “wide” angle (*M*. *orientalis*). From among all the genetically identified females, 94% of *M*. *orientalis* and 97% of *M*. *intestinalis* females were correctly identified by either using the direction of the dorsolateral thoracic protuberances or the angle between caudal rami and the anteroposterior axis.

#### Discriminant function analysis for males

In total, 90 males were entered into the DFA with the following prior probabilities per group: *M*. *intestinalis* 0.51 (n = 46) and *M*. *orientalis* 0.49 (n = 44). For males, one discriminant function was extracted and was highly significant (Wilks’ λ = 0.692, F_1,88_ = 6.152, p < 0.001). The first discriminant function with standardized discrimination coefficients for males is given by (acr = angle between caudal rami and anteroposterior axis):

$$M_{i}=0.76\times protuberances_{i}+0.49\times length_{i}-0.48\times segments_{i}-0.46\times cephalosome_{i}-0.19\times width_{i}-0.06\times acr_{i}$$

For males, the discriminant distributions showed more overlap between the two groups than for females (Fig 7B). This also showed up in the procedure’s classification of the males, which was less reliable than that of the females described above; only 78% of genetically identified *M*. *intestinalis* males and 73% of *M*. *orientalis* males were correctly assigned to one of the two *Mytilicola* groups by the discriminant analysis.

As in females, one variable, the direction of the dorsolateral thoracic protuberances, had the highest coefficient in the discriminant function. Again, the backward selection procedure showed that the direction of the dorsolateral thoracic protuberances was the last variable to be dropped, indicating its importance in the discriminant function. While relying solely on this feature’s direction as morphological identification marker, some males could still be correctly assigned to the genetically identified species, but the success rate was not as high as that for females: 70% of the males of *M*. *orientalis* and 76% of those of *M*. *intestinalis* were correctly identified. The angle between the caudal rami and the anteroposterior axis of the body was less important in discriminating between groups for males than females, but if this feature alone was used as an identification criterion, 64% of the genetically identified *M*. *orientalis* and 63% of the *M*. *intestinalis* males were correctly identified. Among all the genetically identified males, 82% of those of *M*. *orientalis* and 78% of those of *M*. *intestinalis* males were correctly identified by either the direction of the dorsolateral thoracic protuberances or the angle between the caudal rami and anteroposterior axis.

### Effects of preservation and storage method on morphology

We compared two groups of *Mytilicola* spp. that differed in preservation and storage methodology, using fresh specimens of similar size that were either preserved directly in ethanol or frozen prior to ethanol storage. Specimens became significantly smaller after being stored directly in ethanol (all p < 0.05), but the direction of the dorsolateral thoracic protuberances and the number of body segments carrying these protuberances remained the same (Table 2). Length measurements (body length, cephalosome length, body width, genital double-somite length) were, on average, 17.4% shorter after preservation in pure ethanol. In contrast, there was no significant difference in body length between fresh and frozen samples (all p > 0.05), but the latter shrunk significantly more after transfer to ethanol (p < 0.05 for each measurement, except p = 0.106 for genital double-somite length); however, the degree of shrinkage (9.0%) was less than in specimens directly preserved in ethanol (Table 2).

**Table 2:** Effects of preservation and storage methods on morphology of *Mytilicola* spp.

	Fresh → Ethanol				Frozen → Ethanol			
	% shrinkage	t	df	p	% shrinkage	t	df	p
Body length	17.2	2.348	31	< 0.05	7.4	3.059	28	< 0.05
Cephalosome length	22.2	4.112	32	< 0.05	8.3	2.739	28	< 0.05
Body width	14.1	2.624	31	< 0.05	10.9	3.101	28	< 0.05
Genital double-somite length	16.0	2.437	14	< 0.05	9.5	1.709	17	0.106
Mean	17.4				9.0			

Percentage of shrinking of various morphological measurements of *Mytilicola* spp. after storing fresh or frozen samples in ethanol, with significance assessed by Welch’s paired t-tests given in the adjacent columns.

## Discussion

Our analyses show that in our study area, where both invasive species of parasitic copepods now co-occur, *M*. *orientalis* and *M*. *intestinalis* are morphologically differentiated to a significant degree, but with considerable overlap. No macroscopic diagnostic character, or combination of two or three characters, serves to distinguish them with full accuracy. The error rate of assignment to molecular species was more than three times higher in males (25%) than in females (7%). These error rates were obtained by a single observer (MAG) and may therefore represent a best-case scenario.

The present two species of *Mytilicola* were originally described from different parts of the globe (the Mediterranean Sea and Japan) and from different hosts (blue mussels and Pacific oysters). Now that the species' ranges and hosts have come to overlap in northwestern Europe, a sharp distinction between their morphologies is not seen. Whether the native populations of *M*. *orientalis* and *M*. *intestinalis* are more clearly differentiated morphologically would be interesting to find out but is unknown at present. A possible explanation for overlapping morphologies between species in sympatry is that they hybridise (co-occurrence within a host has been observed by Goedknegt et al. [26]); however, our nuclear DNA TaqMan assay detected no hybrids among 75 individuals from the present study (consisting of 35 individuals morphologically identified as *M*. *intestinalis*, and 40 as *M*. *orientalis*). Hybridisation thus appears not to be the cause for overlapping morphologies in the present instance.

For both males and females, the direction of the dorsolateral thoracic protuberances proved to be the most important discriminant factor in the multivariate analyses. Using only the direction of the dorsolateral thoracic protuberances for identification, the two species can still be correctly distinguished in many cases (females, 87–97%; males, 70–76%). The direction of the dorsolateral thoracic protuberances (folded inwards or extended outwards) is generally easier to observe in females as females of *M*. *orientalis* carry extended dorsolateral thoracic protuberances on almost every segment of the body whereas in males only the third and fourth body segments are extended [24] (unpublished observations by MAG). While this is a qualitative feature that is subject to personal observation bias, it has nonetheless been used by several authors up to now [15, 18, 19].

Another straightforward morphological feature, the angle between the caudal rami and the anteroposterior axis, has been mentioned previously as a useful distinguishing feature [18, 19], but in the present study this variable proved useful only for discriminating between females of the two species. Other morphological measurements such as body length and width and the lengths of the cephalosome and (females only) genital double-somite did not prove useful in discriminating between species in the discriminant analysis. Interestingly though, some of these features appeared to be related to the place of origin or the host of the parasites. Copepod body length was significantly related to location (but only for parasites originating from oysters), host size (only for parasites originating from mussels, although size is less easily measured in the irregularly shaped oysters, thus possibly obscuring any parasite size–host size relationship) and host species (only for *M*. *orientalis*). Because of collinearity, other body size measurements are likely related in the same way. If so, a series of more comprehensive morphological measurements will not increase the reliability of morphological identification. Besides measurements per se, the number of body segments carrying dorsolateral thoracic protuberances is not an important identification feature as it only discriminates between sexes (and life stages) [39]. In sum, among all the examined macroscopic morphological variables, the direction of the dorsolateral thoracic protuberances and, to a lesser extent, the angle between the caudal rami and the anteroposterior axis can be considered the most reliable and feasible identification features.

The fact that both parasite species were somewhat differentiated morphologically between locations and hosts indicates that at local scale genetic drift can lead to subtle difference in morphology even while stabilizing selection over larger time sales maintains the overall morphological similarity between species.

For practical applications, our results suggest that initial preservation and storage of *Mytilicola* specimens either in ethanol or by freezing can be recommended if species identification in this genus is to be based on the direction of the dorsolateral thoracic protuberances, because this feature is not affected by the mode of preservation or manner of storage. However, preservation and storage of fresh samples in ethanol is advisable for molecular identification. If morphological measurements are intended, shrinkage in body size (particularly with respect to the length and width of the body and the lengths of the cephalosome and genital double-somite) must be taken into account compared to fresh samples. Our study indicates that shrinkage can be reduced if samples are first frozen and then transferred to ethanol. Regarding species identification, the method of choice will depend on the level of accuracy required for the research question at hand. If 100% accuracy is needed, then identification with molecular methods will be required for both sexes. However, if lower accuracy is acceptable, identification can be based on the direction of the dorsolateral thoracic protuberances leading to 87–97% and 70–76% accuracy in females and males, respectively. Alternatively, as the error rate of especially females is quite low and individuals of both sexes co-occur simultaneously in one host [26], future monitoring surveys may also be based on the morphological examination of females alone. Nevertheless, as these data were collected by one experienced observer, the reliability of morphological identification is likely to vary among multiple and/or unexperienced observers.

## Conclusions

No macroscopic diagnostic character that can be checked by relatively fast and simple morphological observations can be used to reliably discriminate between the two co-occurring invasive parasite species *Mytilicola orientalis* and *M*. *intestinalis* in their invaded range in northwest Europe. This is a good example of how the identification of morphologically similar invaders can be compromised. Even when one invader is known to occur in an ecosystem, the presence of morphologically similar relatives can be missed, making the latter “cryptic invaders”. More attention should be paid to the potential existence of cryptic invasions of parasites. When site of collection and/or host species is no longer operationally discriminative between similar species, a closer examination of potential morphological and genetic means of differentiating them may become necessary. In the present case, molecular identification proved more reliable than morphological species determination, but this need not always to be true.

## Supporting information

S1 Fig Pair plot for all morphological measurements of *Mytilicola spp*. including the morphological variables body length, body width, cephalosome length, angle between caudal rami and anteroposterior axis (acr), and length of the genital double-somite (gds length). The lower diagonal elements contain the (absolute) correlations. Collinearity was especially shown for body length and other morphological measurements.
(DOCX)

S2 Fig Diagnostic restriction fragment assay for *Mytilicola intestinalis* and *M*. *orientalis*. Lanes 1–8: *M*. *intestinalis* (diagnostic bands 366 and 217 bp, indicated by black arrows in lane 1); lanes 9–14, 16 and 17: *M*. *orientalis* (diagnostic bands 286, 222 and 75 bp; indicated by black arrows in lane 10; the 75 bp band is usually faint); lane 15: ambiguous pattern showing bands from both species; traces of incompletely digested DNA are often present; unlabelled lanes are intact PCR products before restriction; sizing ladders consist of bands from 100 to 1000 bp spaced by 100 bp intervals.
(DOCX)

S1 Table Background information on sampled *Mytilicola* spp. including regions of sampling locations, coordinates for each location and the month and year of host collection. (DOCX)

S1 Appendix Raw data used for the multivariate analyses (PCA and DFA). (TXT)

S2 Appendix Raw data used for the preservation and storage method analysis. (TXT)
