# Supplementary material for: Cryptic invasion of a parasitic copepod: Compromised identification when morphologically similar invaders co-occur in invaded ecosystems
Source: PLoS One. 2018 Mar 14;13(3):e0193354. doi: 10.1371/journal.pone.0193354 (PMC5851579; doi:10.1371/journal.pone.0193354)
Supplement: S1 Fig — The lower diagonal elements contain the (absolute) correlations. Collinearity was especially shown for body length and other morphological measurements. (DOCX) [file pone.0193354.s001.docx]

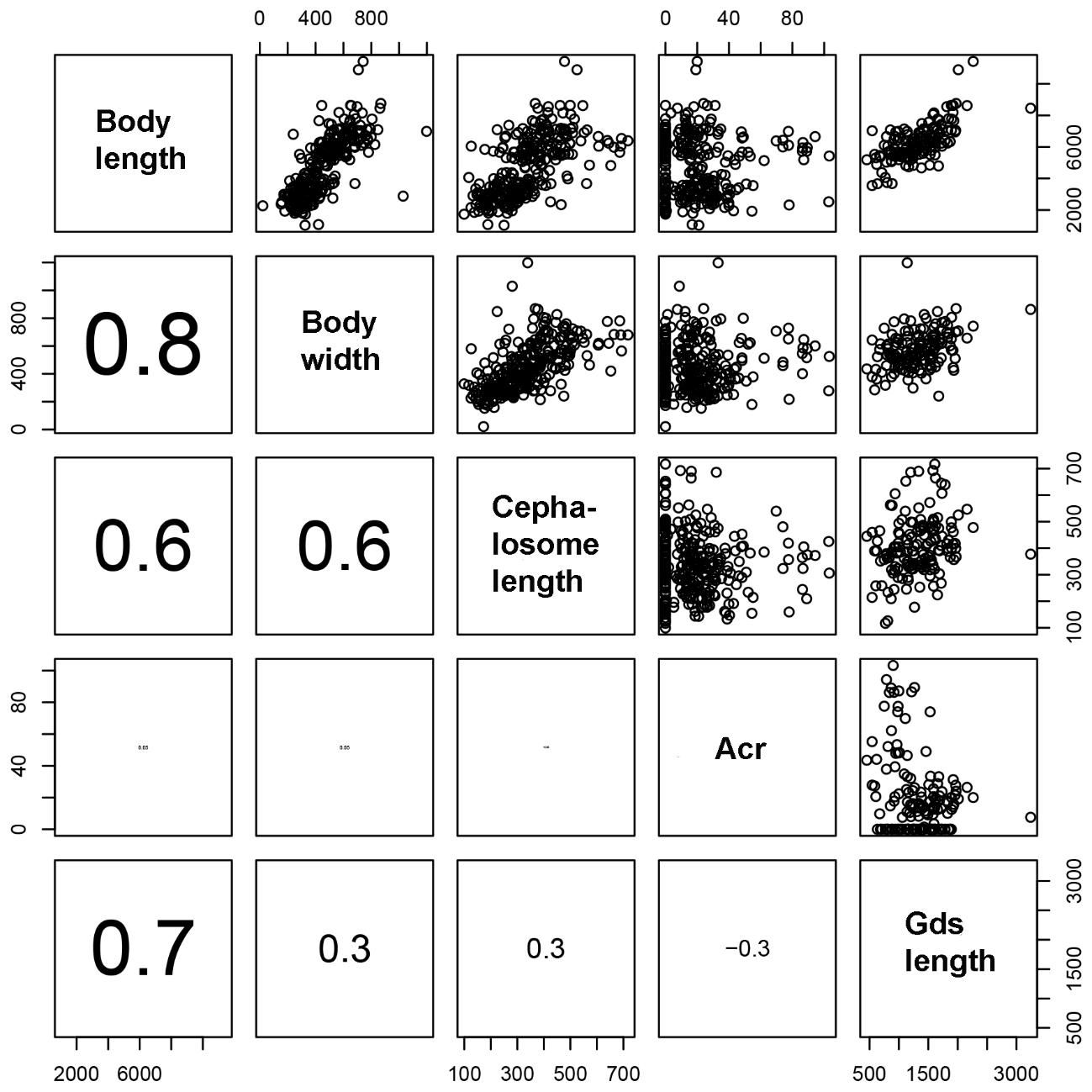


**S1 Fig.** **Pairplot for all morphological measurements of *Mytilicola spp..*** Including the morphological variables body length, body width, cephalosome length, angle between caudal rami and anteroposterior axis (acr), and length of the genital double-somite (gds length). The lower diagonal elements contain the (absolute) correlations. Collinearity was especially shown for body length and other morphological measurements.
