# Supplementary material for: Cryptic invasion of a parasitic copepod: Compromised identification when morphologically similar invaders co-occur in invaded ecosystems
Source: PLoS One. 2018 Mar 14;13(3):e0193354. doi: 10.1371/journal.pone.0193354 (PMC5851579; doi:10.1371/journal.pone.0193354)
Supplement: S2 Fig — Lanes 1–8: M. intestinalis (diagnostic bands 366 and 217 bp, indicated by black arrows in lane 1); lanes 9–14, 16 and 17: M. orientalis (diagnostic bands 286, 222 and 75 bp; indicated by black arrows in lane 10; the 75 bp band is usually faint); lane 15: ambiguous pattern showing bands from both species; traces of incompletely digested DNA are often present; unlabelled lanes are intact PCR products before restriction; sizing ladders consist of bands from 100 to 1000 bp spaced by 100 bp intervals. (DOCX) [file pone.0193354.s002.docx]

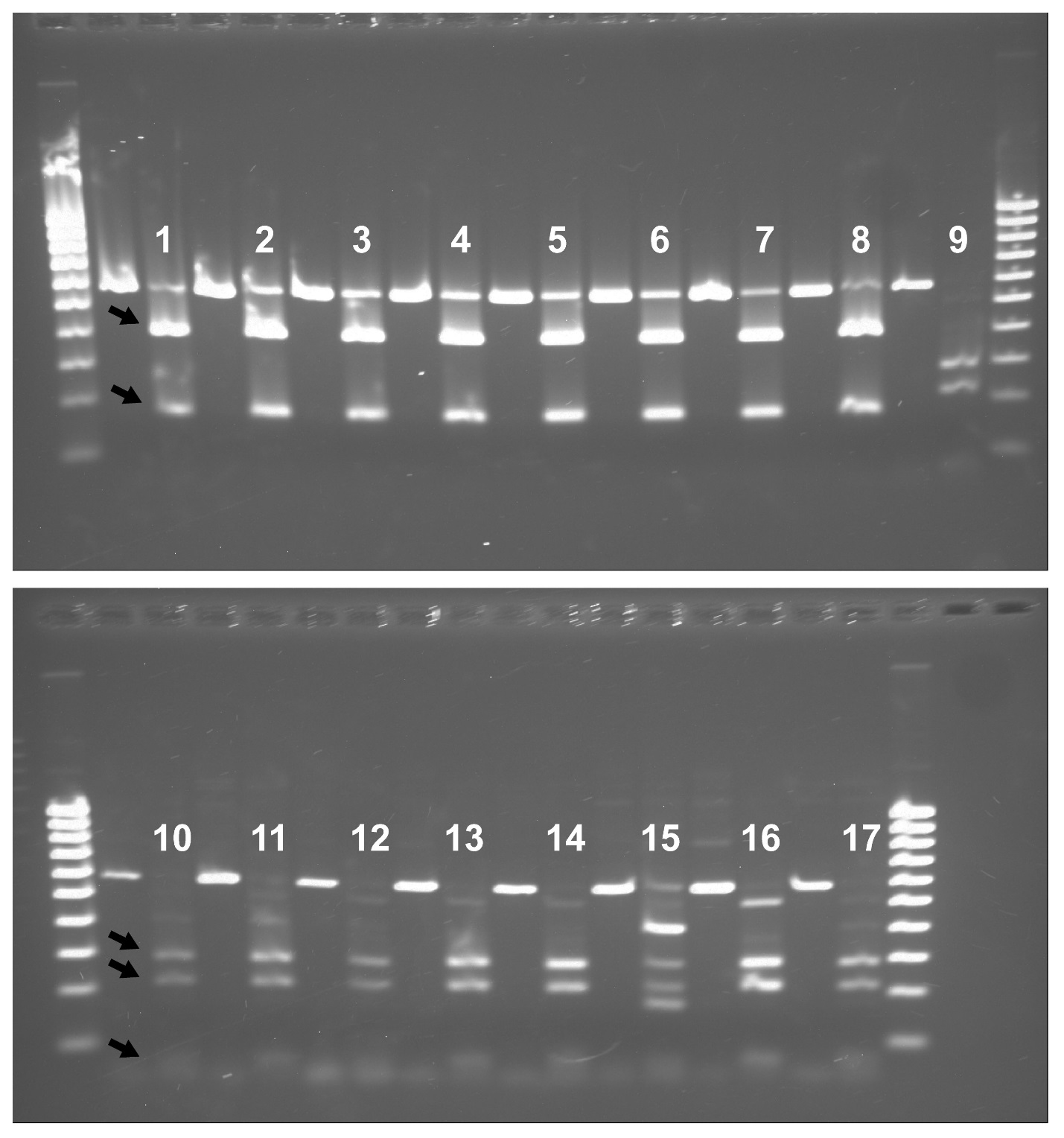


**S2 Fig. Diagnostic restriction fragment assay for *Mytilicola intestinalis* and *M. orientalis*.** Lanes 1-8: *M. intestinalis* (diagnostic bands 366 and 217 bp, indicated by black arrows in lane 1); lanes 9-14, 16 and 17: *M. orientalis* (diagnostic bands 286, 222 and 75 bp; indicated by black arrows in lane 10; the 75 bp band is usually faint); lane 15: ambiguous pattern showing bands from both species; traces of incompletely digested DNA are often present; unlabelled lanes are intact PCR products before restriction; sizing ladders consist of bands from 100 to 1000 bp spaced by 100 bp intervals.
