# Supplementary material for: Cryptic invasion of a parasitic copepod: Compromised identification when morphologically similar invaders co-occur in invaded ecosystems
Source: PLoS One. 2018 Mar 14;13(3):e0193354. doi: 10.1371/journal.pone.0193354 (PMC5851579; doi:10.1371/journal.pone.0193354)
Supplement: S1 Table — (DOCX) [file pone.0193354.s003.docx]

# S1 Table. Background information on sampled *Mytilicola spp*.. Including regions of sampling locations, coordinates for each location and the month and year of host collection.

| **Region** | **Location** | **Coordinates** | **Date** |  |
| --- | --- | --- | --- | --- |
| **Wadden Sea** | 1 | 55.0175 ºN, 8.2605 ºE | Sept 2010/May 2012 | |
|  | 2 | 54.4748 ºN, 8.1841 ºE | May 2012 | |
|  | 3 | 53.0951 ºN, 4.5332 ºE | August 2010 | |
|  | 4 | 53.0858 ºN, 4.5427 ºE | May 2012 | |
|  | 5 | 53.0646 ºN, 4.5434 ºE | February 2011 | |
|  | 6 | 53.0041 ºN, 4.5903 ºE | March 2011 | |
|  | 7 | 52.5580 ºN, 4.5412 ºE | March 2011/May 2012 | |
|  | 8 | 52.5605 ºN, 4.4888 ºE | November 2010 | |
|  | 9 | 53.0022 ºN, 4.4541 ºE | May 2012 | |
| **Dutch Delta** | 10 | 51.4048 ºN, 8.1179 ºE | May 2012 | |
|  | 11 | 51.4017 ºN, 4.0617 ºE | May 2012 | |
|  | 12 | 51.3743 ºN, 3.5507 ºE | May 2012 | |
|  | 13 | 51.4121 ºN, 3.4723 ºE | May 2012 | |
|  | 14 | 51.3729 ºN, 3.4213 ºE | May 2012 | |
|  | 15 | 51.3141 ºN, 3.5824 ºE | May 2012 | |
